# Potential predictive value of circulating tumor DNA (ctDNA) mutations for the efficacy of immune checkpoint inhibitors in advanced triple-negative breast cancer

**DOI:** 10.3389/fgene.2023.1125970

**Published:** 2023-03-16

**Authors:** Qiaorui Tan, Yajing Chi, Mu Su, Jinxing Zhou, Dongdong Zhou, Fangchao Zheng, Xiaochu Man, Shujuan Sun, Jie Huang, Huihui Li

**Affiliations:** ^1^ Department of Medical Oncology, Shandong Cancer Hospital and Institute, Shandong First Medical University and Shandong Academy of Medical Sciences, Jinan, Shandong, China; ^2^ School of Medicine, Nankai University, Tianjin, China; ^3^ Berry Oncology Corporation, Beijing, China

**Keywords:** triple-negative breast cancer, immune checkpoint inhibitors, ctDNA, next-generation sequencing, efficacy

## Abstract

**Background:** In recent years, tumor immunotherapy has become a viable treatment option for triple negative breast cancer (TNBC). Among these, immune checkpoint inhibitors (ICIs) have demonstrated good efficacy in advanced TNBC patients with programmed death-ligand 1 (PD-L1) positive expression. However, only 63% of PD-L1-positive individuals showed any benefit from ICIs. Therefore, finding new predictive biomarkers will aid in identifying patients who are likely to benefit from ICIs. In this study, we used liquid biopsies and next-generation sequencing (NGS) to dynamically detect changes in circulating tumor DNA (ctDNA) in the blood of patients with advanced TNBC treated with ICIs and focused on its potential predictive value.

**Methods:** From May 2018 to October 2020, patients with advanced TNBC treated with ICIs at Shandong Cancer Hospital were included prospectively. Patient blood samples were obtained at the pretreatment baseline, first response evaluation, and disease progression timepoints. Furthermore, 457 cancer-related genes were evaluated by NGS, and patients’ ctDNA mutations, gene mutation rates, and other indicators were determined and coupled with clinical data for statistical analysis.

**Results:** A total of 11 TNBC patients were included in this study. The overall objective response rate (ORR) was 27.3%, with a 6.1-month median progression-free survival (PFS) (95% confidence interval: 3.877–8.323 months). Of the 11 baseline blood samples, 48 mutations were found, with the most common mutation types being frame shift indels, synonymous single-nucleotide variations (SNVs), frame indel missenses, splicing, and stop gains. Additionally, univariate Cox regression analysis revealed that advanced TNBC patients with one of 12 mutant genes (CYP2D6 deletion and GNAS, BCL2L1, H3F3C, LAG3, FGF23, CCND2, SESN1, SNHG16, MYC, HLA-E, and MCL1 gain) had a shorter PFS with ICI treatment (*p* < 0.05). To some extent, dynamic changes of ctDNA might indicate the efficacy of ICIs.

**Conclusion:** Our data indicate that ICI efficacy in patients with advanced TNBC may be predicted by 12 mutant ctDNA genes. Additionally, dynamic alterations in peripheral blood ctDNA might be used to track the effectiveness of ICI therapy in those with advanced TNBC.

## 1 Introduction

Breast cancer has become the leading cause of cancer-related morbidity and mortality in women worldwide (Bray et al., 2018; Feng et al., 2019; Siegel et al., 2021). Triple negative breast cancer (TNBC) accounts for 12%–17% of all breast cancer cases, is commonly recurrent and metastatic, and has a median overall survival (OS) of only 10–13 months (William D Foulkes et al., 2010; Schumacher and Schreiber, 2015; Garrido-Castro et al., 2019). TNBC is the most drug resistant subtype of breast cancer because of low or absent expression of the estrogen receptor (ER), progesterone receptor (PR), and human epidermal growth factor receptor 2 (HER2) (Adams et al., 2019). Despite chemotherapy remaining the most effective course of treatment, advanced TNBC patients usually have limited response to chemotherapy and develop disease progression. Therefore, it is critical to continuously examine therapeutic efficacy and seek out new treatment options.

TNBC differs from other subtypes of breast cancer in various ways, including having a higher mutation frequency, higher percentage of tumor-infiltrating lymphocytes, and 20% of TNBC cells expressing programmed death-ligand 1 (PD-L1) (Mittendorf et al., 2014; Budczies et al., 2015; Schumacher and Schreiber, 2015; Luen et al., 2016). Because of these characteristics, immune checkpoint inhibitors (ICIs) may have a higher efficacy in TNBC than in other breast cancer subtypes. To date, clinical trials have tested a range of ICIs, both independently and in combination, such as programmed cell death protein 1 (PD-1)/PD-L1 inhibitors plus chemotherapy. In the Impassion 130 study, patients with advanced TNBC who were PD-L1 positive and treated with atezolizumab and nab-paclitaxel had prolonged progression-free survival (PFS) and OS rates (Schmid et al., 2018; Iwata et al., 2020; Chen et al., 2022). Similar findings were reported in the KEYNOTE-355 study, which found that pembrolizumab in combination with chemotherapy significantly improved OS in PD-L1 positive patients with advanced TNBC compared with chemotherapy alone (Cortes et al., 2020; Cortes et al., 2022). However, ICIs are not appropriate for all advanced TNBC patients. The KEYNOTE-119 study found that in advanced TNBC patients, pembrolizumab did not significantly prolong OS compared with chemotherapy alone (Winer et al., 2021). Similarly, the IMpassion 131 study discovered that advanced TNBC patients who received paclitaxel with atezolizumab did not display significantly improved PFS or OS (Miles et al., 2021). These results suggest that current ICIs treatment strategies may not be applicable to all advanced TNBC patients. Therefore, it is necessary to identify specific biomarkers for ICI treatment effectiveness to help determine which advanced TNBC patients could possibly benefit from such therapies.

Currently, the need for real-time illness surveillance cannot be satisfied by traditional diagnostic methods like CT imaging (Zhang et al., 2020). Interestingly, there is occasionally a genetic divergence between the primary tumor cells and metastatic breast cancer cells (Itakura et al., 2015). Circulating tumor DNA (ctDNA) is DNA that has been released to blood by primary and metastatic cancer cells during apoptosis and necrosis (Swarup and Rajeswari, 2007). Compared with traditional biopsy methods, examining ctDNA levels offers unique advantages. For example, ctDNA-based approaches overcome tumor heterogeneity and provide real-time molecular data on driver genes, drug resistance genes, and clonal organization (Abstracts of Presentations at the, 2022; Sant et al., 2022). Additionally, lesions that are difficult to evaluate using imaging can be analyzed (Appierto et al., 2017; Hrebien et al., 2019). These ctDNA-based liquid biopsies, which elucidate genetic markers in patient plasma using next-generation sequencing (NGS), can supplement the current screening techniques and have proven to be a powerful option for monitoring cancer progression (Heitzer et al., 2019; Pantel and Alix-Panabieres, 2019). Research on accurate breast cancer diagnosis and treatment has also recently utilized ctDNA. Multiple studies in early breast cancer discovered that ctDNA clearance was related to improved survival after neoadjuvant treatment (Radovich et al., 2020; Magbanua et al., 2021; Ortolan et al., 2021). Additionally, Chen et al. found that ctDNA mutations offer useful information for evaluating the effectiveness of targeted therapies in breast cancer patients who are resistant to trastuzumab and chemotherapy (Chen et al., 2020). Furthermore, their findings demonstrated that gene amplifications and decreased ctDNA levels were related to higher PFS in patients with advanced TNBC after chemotherapy (Wongchenko et al., 2020; Collier et al., 2021). However, there is a lack of studies predicting the efficacy of ICIs in advanced TNBC through ctDNA detected by NGS.

In this study, we aim to identify novel biomarkers that predict the responses to ICI treatments and explore the patterns of ctDNA mutation changes in relation to immunotherapy efficacy by analyzing the ctDNA modifications and mutations in plasma of advanced TNBC patients during ICI therapy.

## 2 Materials and methods

### 2.1 Patients and samples

All patients were recruited prospectively from Shandong Cancer Hospital and Institute, Jinan, Shandong, China, from May 2018 to October 2020. The study was approved by the Institutional Review Board and the Ethics Committee of Shandong Cancer Hospital and Institute. Eligible cases had progressed TNBC, which was characterized by ER and PR immunohistochemistry (IHC) staining of less than 1% of tumor cell nuclei, a HER2 IHC score of 0-1 or 2+, and fluorescence *in situ* hybridization was negative. Pathological examinations were performed by the pathologists in the Department of Pathology, Shandong Cancer Hospital and Institute.

**FIGURE 1:**
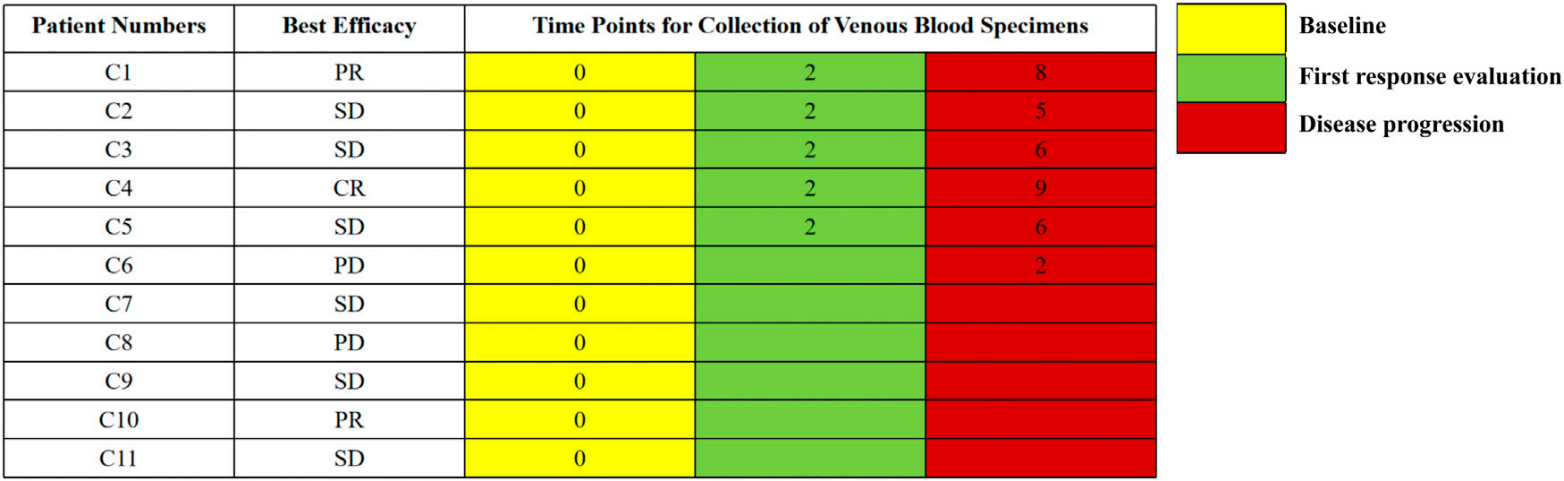
The timepoints for collection of venous blood specimens. The numbers represent the cycles of treatment.

All eligible patients provided written informed consent before participating in this study. Every two cycles of ICI treatments, patients were evaluated for a response using the Response Evaluation Criteria in Solid Tumors (RECIST) 1.1 criteria. Patients’ reactions to ICIs were classified as complete response (CR), partial response (PR), stable disease (SD), or progressive disease (PD) using CT scans and the RECIST 1.1 criteria. We collected dynamic peripheral blood specimens from the patients at baseline, first efficacy assessment, and disease progression. All eligible patients had baseline blood specimens and six patients had dynamic blood specimens (Figure 1). The last follow-up appointment was 29 January 2021. At the time of the final follow-up, all eligible patients were still alive. All relevant clinical data were obtained by reviewing patients’ medical records and contacting them over the phone. The time from registration to illness progression was defined as PFS.

### 2.2 Plasma isolation and ctDNA library construction

Peripheral blood samples were collected in special cell-free DNA BCT tubes (Streck Laboratories, United States). Plasma supernatant and blood cell sediment were separated after whole blood samples were centrifuged at 4°C for 10 min (1,600 x g), then the process was repeated (16,000 x g). The ctDNA was isolated using the MagMAX Cell-Free DNA Isolation Kit (Thermo Fisher Scientific, United States) according to the manufacturer’s instructions. Gel electrophoresis and a Qubit^®^ 4.0 Fluorometer (Life Technologies, United States) were used to quantify the purity of the isolated DNA. The pre-libraries were created using the previously described technique for targeted sequencing of ctDNA (Lv et al., 2015). To create a sequencing library, ctDNA fragments were collected using the NGS gene panel, which includes 457 cancer-associated genes (Berry Oncology, Peking, China) (Supplementary Table S1). The Illumina NovaSeq 6000 platform (Illumina, San Diego, CA, United States) was used to apply the sequencing libraries in 150PE mode.

### 2.3 Bioinformatics analysis of ctDNA mutations

FASTP (Chen et al., 2018) was used to trim adapters and obtain clean results by removing low-quality sequences. By using Burrows-Wheeler Alignment tool (BWA), the clean reads were aligned to the Ensemble GRCh37/hg19 reference genome (Li and Durbin, 2009). PCR duplications were processed by gencore (Chen et al., 2019), then consensus reads were generated. SAMtools (Li et al., 2009) was applied for the detection of single-nucleotide variations (SNVs), insertions, and loss. The ANNOVAR software (Wang et al., 2010) was used to annotate the HGVS variant description. After annotation, we eliminated non-synonymous SNVs with PopFreqMax >0.05 and retained those with VAF >0.5% or >0.1% in cancer hotspots obtained from patient databases for further investigation. Tumor mutation burden (TMB) was defined as non-synonymous coding mutations per Megabase. For copy number variants (CNVs), the gene was regarded as gain when the copy number >3 and regarded as loss when the copy number <1.

### 2.4 Statistical analysis

R version 3.4.0 was used for all analyses. Survival-related ctDNA was identified using univariate and multivariate Cox regression analyses. Kaplan-Meier (KM) survival curve differences were assessed by log-rank analysis. Fisher’s exact test was performed to compare the ctDNA variations among patients with advanced TNBC. Each test was two-sided, and*P*-values <0.05 were considered significant.

## 3 Results

### 3.1 Characteristics of enrolled patients

Using the eligibility criteria, 11 advanced TNBC patients who received ICIs were enrolled in this study. Among these 11 patients, the median age at diagnosis was 46 years (range 34–67 years). Most patients (54.5%, 6/11) were diagnosed with stage III disease, while 27.3% (3/11) and 18.2% (2/11) of patients were stage II and stage I, respectively. According to the pathological classification, 10 individuals were grade III, while just one patient was grade II. Additionally, 81.8% (9/11) of patients were diagnosed with invasive ductal carcinoma. The median PFS for the 11 patients was 6.1 months (95% confidence interval (CI): 3.877–8.323 months). All eligible patients received a combination treatment of ICIs plus chemotherapy as first-line treatment (63.6%, 7/11), second-line treatment (18.2%, 2/11), and third-line treatment (18.2%, 2/11). During the treatment procedure, the best efficacy was CR in one case (18.2%), PR in two cases (18.2%), and SD in six cases (54.5%). The clinicopathologic characteristics of the patients are shown in Table 1.

**TABLE 1:** Clinical and pathological features of enrolled patients (N = 11).

Clinical and pathological features		n (%)
Age		
	<50 yearss	6 (54.5%)
	≥50 years	5 (45.5%)
Clinical stage		
	Ⅰ	2 (18.2%)
	Ⅱ	3 (27.3%)
	Ⅲ	6 (54.5%)
Invasive ductal carcinoma		
	Yes	9 (81.8%)
	No	2 (18.2%)
Pathologic grade		
	Ⅱ	1 (9.1%)
	Ⅲ	10 (90.9%)
Lines of therapy		
	1	7 (63.6%)
	2	2 (18.2%)
	3	2 (18.2%)
Treatment regimens		
	PD-1 inhibitor^a^ + nab-paclitaxel	7 (63.6%)
	PD-1 inhibitor + platinum + paclitaxel	1 (9.1%)
	PD-1 inhibitor + capecitabine	1 (9.1%)
	PD-1 inhibitor + platinum + gemcitabine	1 (9.1%)
	PD-L1 inhibitor^b^ + paclitaxel	1 (9.1%)
Best efficacy		
	CR	1 (9.1%)
	PR	2 (18.2%)
	SD	6 (54.5%)
	PD	2 (18.2%)

^a^ PD-1 inhibitor: Toripalimab, Tislelizumab, Sintilimab.

^b^ PD-L1 inhibitor: Atezolizumab.

### 3.2 Mutant genes in ctDNA may predict the efficacy of ICIs

A total of 11 baseline blood specimens were analyzed by NGS, with 457 genes detected (Supplementary Table S1). We observed 48 genetic variants in the 11 baseline blood specimens, with the mutation types primarily including frame shift indels, synonymous SNVs, frame indel missenses, splicing, and stop gains. Within the SNVs, the most frequently mutated gene was TP53 (73%, 8/11), with the other SNV frequencies<20%.

**FIGURE 2:**
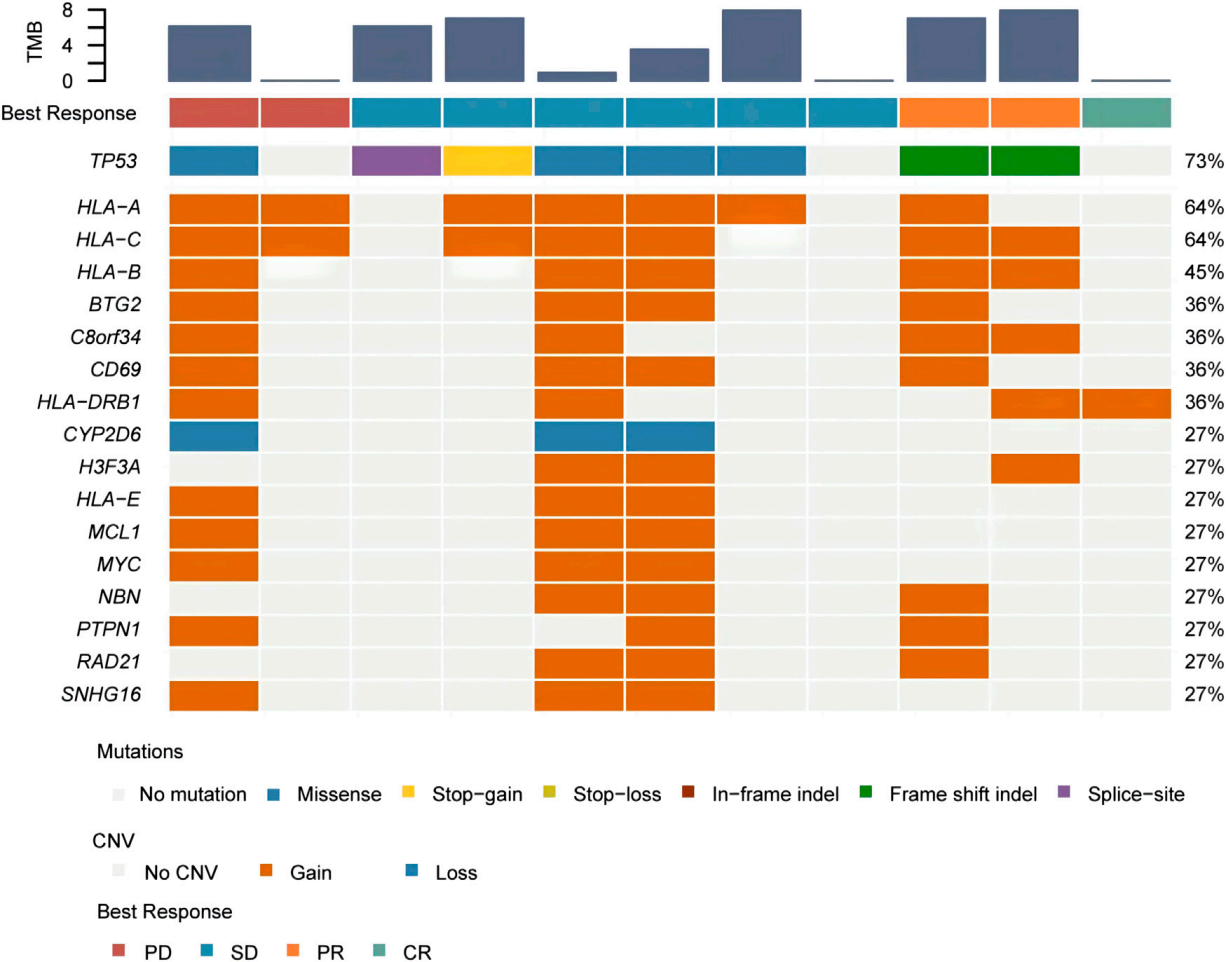
Genes with a mutation frequency greater than 20%.

**FIGURE 3:**
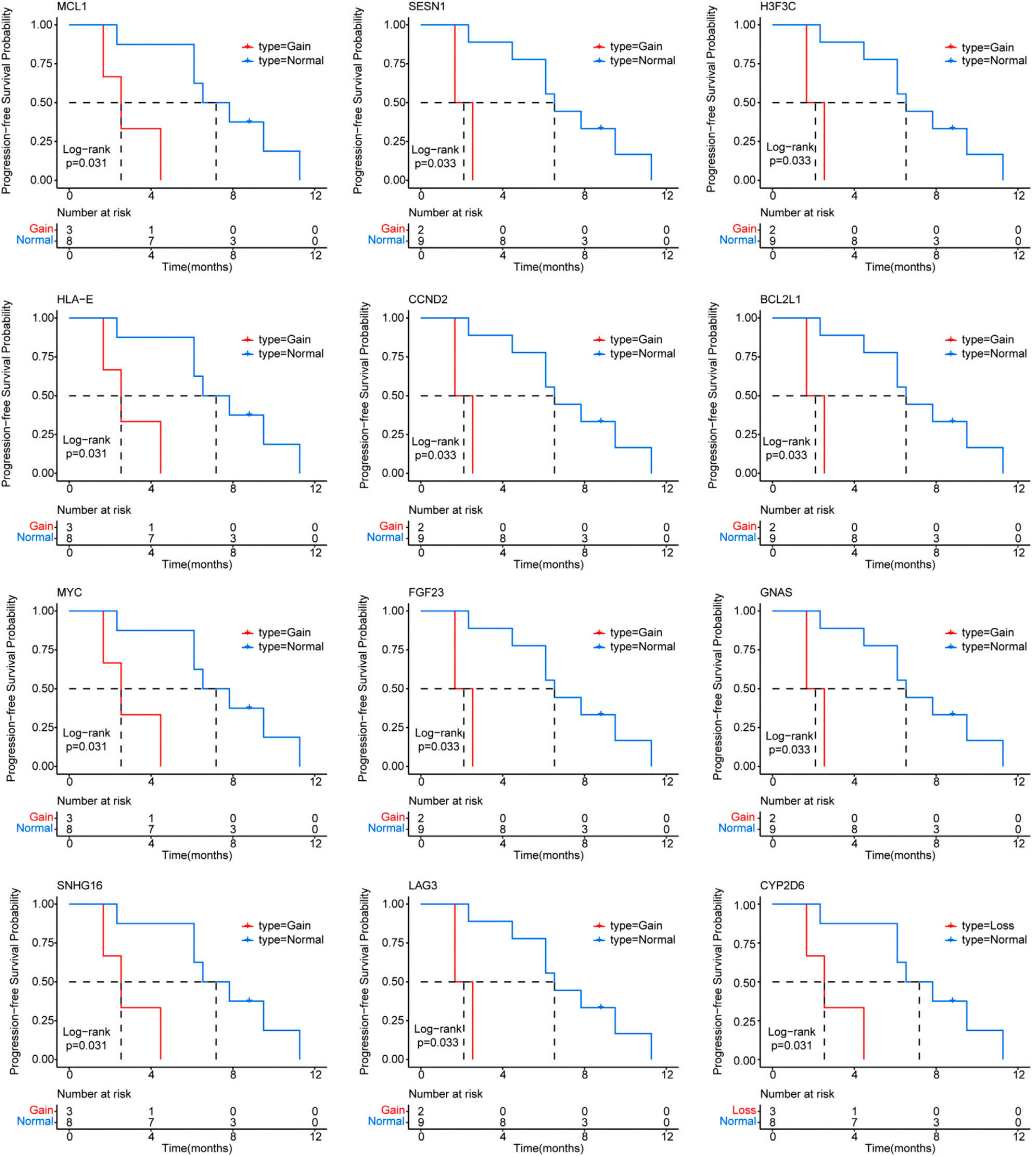
Correlation of circulating tumor DNA (ctDNA) gene mutations with progression-free survival (PFS) in advanced triple negative breast cancer (TNBC) patients receiving immune checkpoint inhibitor (ICI) treatment.

Furthermore, we identified CNVs with mutation frequencies>20% (Figure 2) and found that 64% of patients have HLA-A and HLA-C gain. HLA-B gain and CYP2D6 loss were present in 45% and 27% of patients, respectively. In addition, 27% of patients have HLA-E, MCL1, MYC, NBN, PTPN1, RAD21, and SNHG16 gain. The predictive power of ctDNA in ICI treatment management was then assessed. By univariate Cox regression analysis, we found that CYP2D6 loss and GNAS, BCL2L1, H3F3C, LAG3, FGF23, CCND2, SESN1, SNHG16, MYC, HLA-E, and MCL1 gain were associated with short PFS (*p* < 0.05) (Figure 3). The results indicate that patients with these particular mutations might not fully benefit from ICI therapy. However, multivariate Cox regression analysis did not identify statistically significant mutated genes associated with PFS.

### 3.3 Dynamic changes of mutant genes in ctDNA during ICI treatment

**FIGURE 4:**
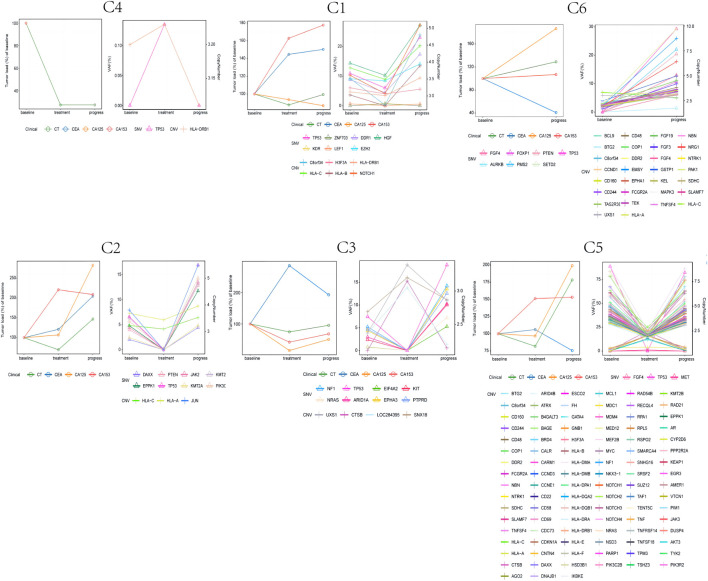
The dynamic changes of circulating tumor DNA (ctDNA) features (copy number variants (CNVs) and single-nucleotide variations (SNVs)), tumor burden, and CEA, CA125, and CA153 levels in six triple negative breast cancer (TNBC) patients.

Considering that ctDNA genomic traits change dynamically with therapy (Garcia-Murillas et al., 2015), we analyzed ctDNA collected from six patients with dynamic blood samples. We also analyzed the changes in carcinoembryonic antigen (CEA), carbohydrate antigen 125 (CA125), and carbohydrate antigen 153 (CA153) levels (Figure 4).

For patients with the best CR, PR, or SD efficacy evaluations, we observed two patterns of ctDNA mutations: 1) the copy number or mutation frequency declined at first response evaluation, while the copy number or mutation frequency rose again at disease progression; 2) the copy number or mutation frequency rose at first response evaluation, while the copy number or mutation frequency declined at disease progression. From these two patterns of mutations, we observed that during ICI treatment, the CNV or SNV initially rises and then falls or initially falls and then rises. When this occurs with changing ctDNA mutations, it possibly indicates that the patients are responding to ICI treatment. Additionally, Patient C6 experienced disease progression at the first response evaluation, at which point the mutation frequency or copy number rose. However, we did not observe this pattern of changes in CEA, CA125 or CA153 associated with ICI efficacy among these six patients.

In future studies, the sample size should be increased and additional blood collection points should be included to further verify the relationship between the dynamic changes of ctDNA and ICI treatment efficacy.

## 4 Discussion

**FIGURE 5:**
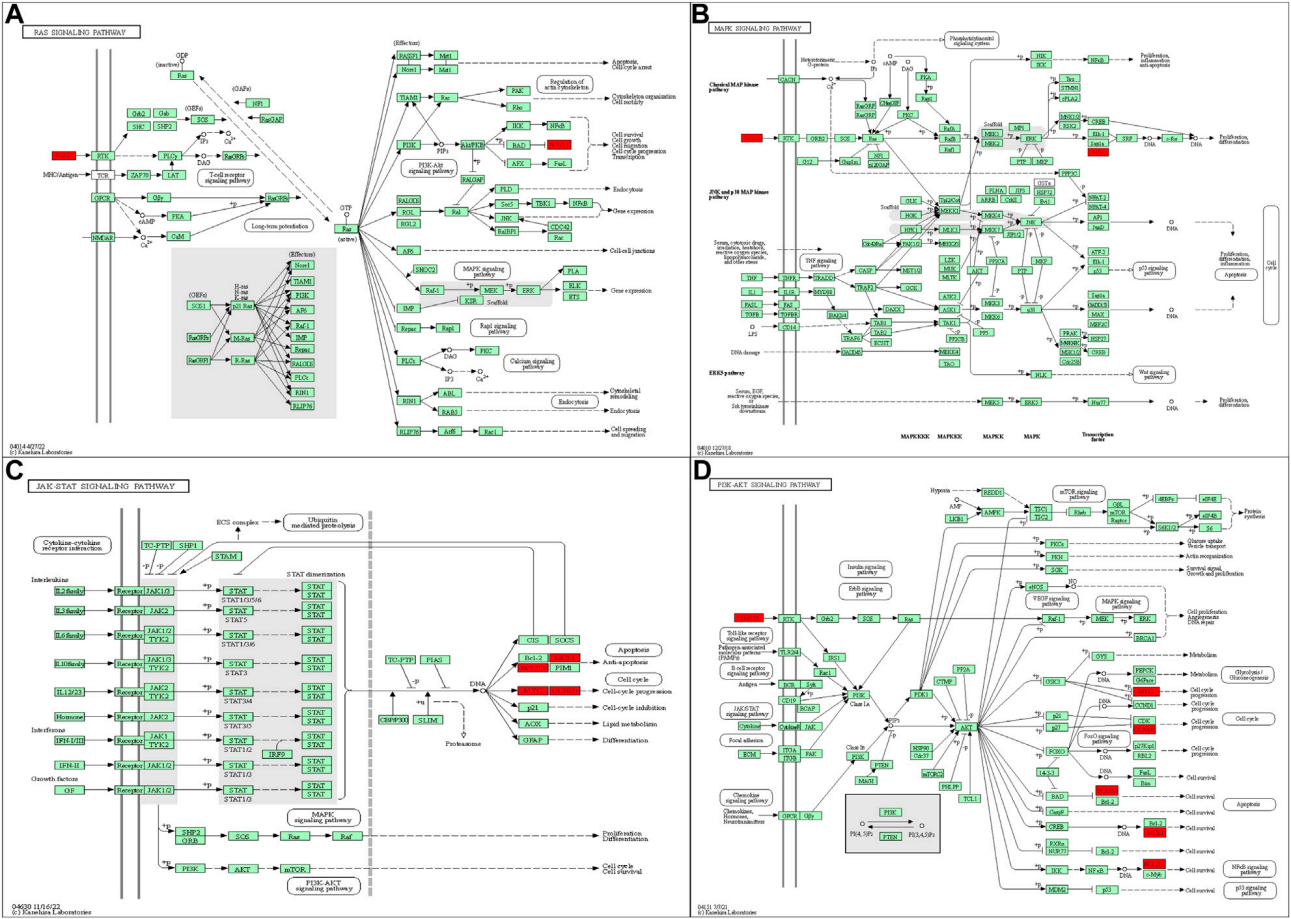
**(A)** FGF23 and BCL2L1 in the RAS signaling pathway. **(B)** FGF23 and MYC in the MAPK signaling pathway. **(C)** MCL1, BCL2L1, MYC, and CCND2 in the JAK-STAT signaling pathway. **(D)** CCND2, FGF23, MCL1, MYC, and BCL2L1 in the PI3K-AKT signaling pathway. All figures are included from the KEGG database.

ICIs are a promising treatment method that has significantly improved TNBC patient treatment outcomes (Cortes et al., 2020; Iwata et al., 2020; Emens et al., 2021; Chen et al., 2022). However, not all patients with advanced TNBC can benefit from ICIs (Miles et al., 2021; Winer et al., 2021), so timely monitoring of ICI efficacy and choosing appropriate patients are essential. Using NGS technology, we dynamically evaluated peripheral blood ctDNA alterations in patients with advanced TNBC during treatment. Concurrent tumor load and tumor marker changes in the included patients were also recorded. We found that 48 genetic mutations occurred in the 11 baseline samples. For the SNV, TP53 was the gene with the highest mutation frequency in advanced TNBC. These results were consistent with previous reports (Jiang et al., 2021; Chen et al., 2022). Furthermore, in advanced TNBC patients who received ICIs, CYP2D6 loss and GNAS, BCL2L1, H3F3C, LAG3, FGF23, CCND2, SESN1, SNHG16, MYC, HLA-E, and MCL1 gain were related to shorter PFS. Our results suggest that advanced TNBC patients with one of these 12 mutated genes are possibly not suitable candidates for ICI treatment. As shown in the literature, BCL2L1, LAG3, CCND2, SNHG16, MYC, HLA-E, MCL-1, and GNAS have regulatory effects on apoptosis, anti-tumor immunity, the tumor cell cycle, cell growth, or metastasis. Among them, BCL2L1 and MCL-1 had an inhibitory effect on apoptosis in TNBC (Kelly and Strasser, 2011; Goodwin et al., 2015). Other studies found that LAG3 and HLA-E were associated with immunosuppressive effects and tumor immune evasion in breast cancer, gastric cancer, and ovarian cancer, among others (Woo et al., 2012; Tuncel et al., 2013; Morandi et al., 2014; Zheng et al., 2015; Su et al., 2016; Andrews et al., 2017; Du et al., 2020). In addition, upregulated CCND2, SNHG16, MYC, and GNAS levels were associated with accelerating cell cycle progression, as well as promoting growth and metastasis in lung cancer, breast cancer, and colon cancer (Cai et al., 2017; Fallah et al., 2017; Teng et al., 2017; Batistatou et al., 2018; Hung et al., 2018; Han et al., 2019; Jin et al., 2019; SM, 2020). Additionally, to explore the linkage among these 12 mutated genes, we utilized the KEGG database to examine the signaling pathways in which these genes are located. We found that several of the mutant genes are implicated in numerous important signaling pathways, both upstream and downstream, including the PI3K-AKT, MAPK, Ras, and JAK-STAT signaling pathways (Christov et al., 2011; Hernandez-Varas et al., 2011; Clinkenbeard et al., 2016; Wang et al., 2017; Luo et al., 2018; Wang et al., 2018; Wu et al., 2019; Zhang et al., 2019; Chang et al., 2020; Schafer et al., 2020; Umehara et al., 2020; Ho and Bergwitz, 2021) (Figure 5). These signaling pathways are involved in the regulation of cancer-related phenotypes, including cell cycle progression, cell survival, cell migration, signal transduction, and more. Therefore, the regulatory interactions between CCND2, FGF23, MCL1, MYC, GNAS, and BCL2L1 may alter the efficacy of ICIs. We hypothesize that the effects of these mutant genes on drug efficacy result from their promoting malignant cellular phenotypes. Future cell experiments will be conducted to confirm this regulatory impact.

Serum CEA, CA153, and CA125 are tumor markers that are frequently used in breast cancer screening and therapy efficacy monitoring (Cheung and Robertson, 2000; Seregni et al., 2004; Duffy, 2006; Duffy et al., 2010). However, traditional tumor markers still have some drawbacks. First, tumor load, physiological and pathological conditions of the body, basic diseases, and medicines can all alter serum tumor marker levels, resulting in lower sensitivity and specificity. Second, the critical value of serum tumor marker levels that insinuate disease progression is unclear (Seregni et al., 2004). Third, 30% of patients who respond to therapy may see a paradoxical increase in tumor marker levels following the start of chemotherapy. This rise could be attributed to the severe neoplastic cell necrosis caused by cytotoxic treatments, and marker levels can remain elevated for up to 3 months (Seregni et al., 2004). Overall, these reasons indicate that traditional tumor markers do not effectively reflect the efficacy of ICIs. Our results suggest that the changes in CEA, CA153, and CA125 levels may not be consistent with the tumor load trends. Because of the small sample size of this study, the connection between traditional tumor markers and ICI efficacy needs to be verified in a larger population.

Currently, previous research has shown that the dynamic changes of ctDNA levels correlate with treatment efficacy in hepatocellular carcinoma, pancreatic cancer, and breast cancer (Cai et al., 2019; van der Sijde et al., 2019; Liu et al., 2022). However, there is a lack of studies on the relationship between ctDNA dynamic changes and ICI efficacy. By monitoring the changes of ctDNA-related mutations at several timepoints during the patient treatment period, we found that some changes in ctDNA were similar to tumor load changes, such as pattern 1. By dynamically monitoring the changes of ctDNA-related mutations during the ICI treatment period of advanced TNBC patients, we found that the elimination of ctDNA mutations or a decreased mutation rate often represented a better ICI efficacy. In contrast, as the disease progressed, the ctDNA mutation pattern revealed that the copy number or mutation frequency rose. However, we also observed that the copy number or mutation frequency rose in patients treated with ICIs despite being effective, such as in pattern 2. Considering the source of ctDNA, we hypothesize that this is the result of chemotherapy effectively triggering tumor cell death. This causes more ctDNA to be released into the blood and allows mutations that were not previously found in the blood to be detected throughout the treatment process (Diaz and Bardelli, 2014). Our results indicate that plasma ctDNA-based liquid biopsy could potentially be used as an additional test to monitor disease in TNBC patients.

In conclusion, our data demonstrate that CYP2D6 loss and GNAS, BCL2L1, H3F3C, LAG3, FGF23, CCND2, SESN1, SNHG16, MYC, HLA-E, and MCL1 gains were predictive of ICI effectiveness in patients with advanced TNBC. In addition, dynamic monitoring of ctDNA in patients with advanced TNBC might provide a timely indicator of sensitivity to ICI treatment. In the future, further studies are necessary to validate our observations.

## Data Availability

The datasets presented in this study can be found in online repositories. The names of the repository/repositories and accession number(s) can be found in the article/Supplementary Material.
